# Phenolic Characterization and Comparative Antioxidant Profiling of Australian *Asparagopsis armata* and *A. taxiformis* Across Their Developmental Stages

**DOI:** 10.3390/antiox15020273

**Published:** 2026-02-23

**Authors:** Kethabile Sonno, Faezeh Ebrahimi, Ziqi Lou, Hoang Chinh Nguyen, Colin J. Barrow, Hafiz A. R. Suleria

**Affiliations:** 1 School of Agriculture, Food and Ecosystem Sciences, Faculty of Science, The University of Melbourne, Parkville, VIC 3010, Australia; kethabileohimile.sonno@student.unimelb.edu.au (K.S.);; 2 Centre for Sustainable Bioproducts, Deakin University, Geelong, VIC 3217, Australia

**Keywords:** Australian macroalgae, *Asparagopsis armata*, *Asparagopsis taxiformis*, ultrasound-assisted extraction, conventional solvent extraction, tetrasporophyte life stage, gametophyte life stage, LC-ESI-QTOF-MS/MS characterization

## Abstract

*Asparagopsis* has gained global attention for its chemical properties and environmental applications. However, its two main species, *Asparagopsis armata* and *Asparagopsis taxiformis*, remain understudied, with limited information available regarding their bioactive potential, especially across their development. In this study, we examined the phenolic profiles and antioxidant potentials of gametophyte and tetrasporophyte life stages and compared differences between conventional solvent extraction (CSE) and ultrasound-assisted extraction (UAE), including total phenol content, total flavonoid content, determination of condensed tannins, and seven types of antioxidant activity detections such as DPPH and ABTS. In general, the phenolic compounds and antioxidant potential of the *Asparagopsis* species vary significantly at different life stages and under different extraction techniques. Among them, the phenolic profile and antioxidant capacity of *A. armata* were recorded as significantly higher than those of *A. taxiformis*, as reflected by its greater relative antioxidant capacity index scores. In our study, while UAE did not universally outperform CSE, species- and life stage-specific improvements were recorded. Moreover, LC-ESI-QTOF-MS/MS tentatively identified 24 phenolic compounds (17 in *A. armata* and 14 in *A. taxiformis)*, pointing to a diverse bioactive profile. Overall, *Asparagopsis* species demonstrated marked variability in phenolic and antioxidant potentials across life stages and extraction techniques.

## 1. Introduction

Seaweed use has been embedded in human culture since ancient times [1]. They have been utilized as a food resource, medicine, livestock feed, fertilizers, and, more recently, in biofuel, pharmaceuticals, nutraceuticals, and functional food development [2]. Worldwide, marine algae production has increased more than threefold, and over 95% of this output had come from cultivation instead of wild collection in 2019 [3]. As of 2019, seaweed farming grossed more than USD 14 billion, with Oceania only contributing to a fraction of the global market [4]. However, the Australian Seaweed Industry is rapidly growing and has been proposed to reach $1.5 billion by 2040, with a profound interest in its native species. Among the leading genera, the Australian *Asparagopsis* has been nominated as the leading opportunity for expansion [5], most likely following the discovery of their potential in reducing enteric methane in ruminants [6], leading to prospective benefits in climate change. Beyond their environmental control potential, this genus has also been studied for other therapeutic capabilities, including nutraceuticals, medicine, and the cosmetics industry [7,8]. Overall, this genus has gained considerable attraction due to its diverse phytochemical content, especially its secondary metabolites.

Secondary metabolites are phytochemical compounds produced in response to biotic and abiotic stressors in the harsh marine environments (UV radiation, temperature, salinity, pollutants, infection, and oxidative stress) and serve as chemical defenses [9]. Notable examples in *Asparagopsis* include halogenated low molecular weight compounds, sterols, carotenoids, and phenolic compounds. For example, Mellouk et al. [10] have observed the total phenolic and fatty acid profiles of the Algerian *A. taxiformis*. Likewise, Pinto et al. [11] have endeavored to record the nonpolar and polar extracts of Azores *A. armata* using GC- and UHPLC-MS and nominated halogenated compounds and fatty acids as the major compounds in this seaweed, with another study identifying metabolites in the Taiwanese *A. taxiformis* using untargeted UHPLC-HRMS and targeted GC-MS [12].

The *Asparagopsis* genus and its bioactive extracts have been linked with numerous benefits, such as antioxidant [13], anti-inflammatory [14], antifouling [15], and anti-acne [7] properties. Moreover, in a 2025 study, the antibacterial and antifungal properties of the Algerian *A. armata* against *Staphylococcus aureus*, *Klebsiella pneumoniae*, *Bacillus cereus*, and select species of *Fusarium* were recorded [16]. Moreover, in an in vitro experiment on the collective influence of phenolic compounds as hydrogen acceptors in the presence of *A. taxiformis* as a ruminal methanogenesis inhibitor, phloroglucinol displayed potential in improving rumen fermentation in dairy goats [17]. However, limited information is available regarding the phenolic makeup of the species, especially in regard to their different life cycles.

Within this genus, two species are more widely recognized: *Asparagopsis armata* Harvey 1855 and *Asparagopsis taxiformis* (Delile) Trevisan 1845. These species differ morphologically, with *A. armata* possessing long stolons that may entangle other marine organisms, as well as the geographical differences [18]. However, both species share a heteromorphic and triphasic life cycle, alternating between a plumose gametophytic (*n*) stage, a microscopic carposporophytic (*2n*) stage formed on the female gametophyte, and a filamentous tetrasporophytic (*2n*) stage [19]. Previous studies have reported differences in the biochemical composition of these species, with the main focus on the gametophyte and tetrasporophyte phases [20,21], although due to the difficulty in sample collection from the female gametophytes and the commercial unavailability of the microscopic carposporophytic stage, this study focused on the two available stages. However, despite such findings, no study has explicitly compared the antioxidant and phenolic contents of these species at distinct life stages, with some studies not considering the life stage.

The current investigation aims to shed light on the possible differences in the antioxidant and phenolic profiles of the *A. armata* and *A. taxiformis* across their developmental stages using various in vitro assays. The influence of extraction methodology will also be assessed, and a comparative phenolic profile between the species and their life stages will be computed using liquid chromatography electrospray ionization quadrupole time-of-flight mass spectrometry (LC-ESI-QTOF-MS/MS). With the rising attention to the *Asparagopsis* genus, this study will pave the way for a better understanding of the metabolic stages and extraction methodologies influencing its possible potentials.

## 2. Materials and Methods

### 2.1. Sample Collection

The four dried samples, *Asparagopsis armata*, tetrasporophyte (AAT), *Asparagopsis armata*, gametophyte (AAG), *Asparagopsis taxiformis*, gametophyte (ATG), *Asparagopsis taxiformis*, tetrasporophyte (ATT), were kindly provided by Sea Forest Pty Ltd. (Triabunna, TAS, Australia).

### 2.2. Extraction Preparation

Seaweed samples were extracted using seaweed sample powder through two extraction techniques: ultrasonic-assisted extraction (UAE) and conventional solvent extraction (CSE). For CSE, 20 mL of 80% ethanol (*v*/*v*) was added to each gram of seaweed powder for each sample and placed in a shaking incubator (ZWYR-240, Labwit, Ashwood, VIC, Australia) for 24 h, with a constant agitation of 210 rpm. For UAE, the same solvent was added to the seaweed samples at the same ratio, with each sample subjected to 3 min of ultrasonication at 60% amplitude using a Qsonica sonicator (Model Q55, Newtown, CT, USA), with samples maintained on ice. Following each extraction, the mixture was centrifuged at 4500× *g* for 15 min, and the supernatant was collected as seaweed extracts, with all results calculated back to the utilized dry weight of seaweeds.

### 2.3. Estimation of Phenolic Content and Antioxidant Activities

To determine the phenolic potential of the *Asparagopsis* samples, total phenolic content (TPC) was measured using the Folin–Ciocalteu and sodium carbonate, using gallic acid as a standard measured at 765 nm, total flavonoid content (TFC) using aluminum chloride and sodium acetate with quercetin as a standard measured at 440 nm, and condensed tannin content using the vanillin–sulfuric acid procedure with catechin standard measured at 500 nm, following the methods described by Siddiqa et al. [22] and Shi et al. [23].

The radical scavenging activities of the extracts against 2,2′-Diphenyl-1-picrylhydrazyl (DPPH) with Trolox standard measured at 517 nm, 2,2′-Azino-bis-3-ethylbenzothiazoline-6-sulfonic acid (ABTS) and potassium persulfate method measured at 734 nm with ascorbic acid standard, and hydroxyl radicals using ferrous sulphate and hydrogen peroxide procedure with an ascorbic acid standard measured at 510 nm were evaluated following the methods described by Duan et al. [24] and Lee et al. [25]. The reducing power of the extracts was analyzed using ferric reducing antioxidant power (FRAP) with sodium acetate, 2,4,6-Tris(2-pyridyl)-s-triazine, and iron chloride method measured at 593 nm with a Trolox standard. Similarly, total antioxidant capacity (TAC) was measured using acidified ammonium molybdate and sodium phosphate with a Trolox standard measured at 695 nm, reducing power assay (RPA) using Trolox as standard measured at 750 nm, and ferrous ion chelating activity (FICA) with EDTA measured at 562 nm, following the previously established protocols described by Imran et al. [26] and Siddiqa, Khaliq, Sultan, Chugthai, Ahsan, Khalid and Suleria [22]. All absorbance values were measured using the Multiskan^®^ Go microplate reader (Thermo Fisher Scientific, Waltham, MA, USA). Results were obtained in triplicate and are expressed as milligrams of standard equivalent per gram sample dry weight (mg std/g dw).

### 2.4. Relative Antioxidant Capacity Index

The relative antioxidant capacity index (RACI) for each sample was calculated based on individual triplicate data points obtained from TPC and antioxidant assays according to the method described by Sun and Tanumihardjo [27]. The final RACI score for each sample was calculated by averaging the z-scores of all data points across the assays.

### 2.5. Phenolic Compound Characterization Using LC-ESI-QTOF-MS/MS Analysis

Phenolic compounds present in the *Asparagopsis* extracts were examined using LC-ESI-QTOF-MS/MS, following the methodology described in [28] with minor modifications. Briefly, protein precipitation was carried out using a methanol:water (80:20, *v*/*v*) ratio of 4:1. Samples were vortexed thoroughly, incubated at 4 °C for 15 min, centrifuged at 12,000× *g* for 10 min, and further diluted to 1:10 with the methanol mixture. The supernatant was filtered through a 0.45 µm syringe filter prior to analysis. The characterization of phenolic compounds was performed using an Agilent 1290 Infinity II HPLC system coupled with an Agilent Revident LC/Q-TOF (Agilent Technologies, Santa Clara, CA, USA). The separation process utilized a Synergi Hydro-RP 80 Å reverse phase column (250 mm × 4.6 mm, 4 μm particle size) (Phenomenex, Lane Cove, NSW, Australia) along with a protected C18 ODS guard column (4.0 × 2.0 mm). The mobile phase A consisted of 99.9% Mili-Q water with 0.1% formic acid, while the mobile phase B comprised 99.9% acetonitrile with 0.1% formic acid at a flow rate of 0.8 mL/min with 1 μL of injection volume. The gradient commenced with 1% B, reaching 2% B at 4 min, increased to 5% B between 4 and 10 min, followed by an increase to 45% B from 10 to 50 min. An escalation to 98% occurred from 50 to 52 min, holding at 98% B for 2 min, before reverting to 2% B from 54 to 56 min, up to 60 min. Peak identification was performed with both positive and negative ionization modes. The Mass Hunter workstation software (Qualitative Analysis, version B.03.01, Agilent Technologies, Santa Clara, CA, USA) and Personal Compounds Database and Library (PCDL) were used for data acquisition and analysis. To ensure accuracy, only compounds with a mass error of ±5 ppm and a PCDL score greater than 80 were considered for *m*/*z* verification and MS/MS identification. Venn diagrams were created using the Venny platform (version 2.1.0) [29], incorporating all compounds that met the acceptable mass-error parameters.

### 2.6. Statistical Analysis

All phenolic measurements and antioxidant activity were analyzed in triplicate, and the data are expressed as mean ± standard deviation (SD). Statistical analysis was carried out using Minitab software (LLC, State College, PA, USA version 18 for Windows) [30], applying a one-way ANOVA and post hoc comparison through Tukey’s HSD test, following a residual normality test [31] using the Ryan–Joiner test (*p* < 0.05) and residual Q–Q plots. If residuals deviated from normality, the SQRT transformation was applied, and assumptions were re-evaluated, with statistical significance defined at *p* < 0.05 (Supplementary Figure S1). Pearson’s correlation coefficient was deployed to analyze correlations between phenolic and antioxidant tests.

## 3. Results and Discussion

### 3.1. Estimation of Phenolic Content

The *Asparagopsis* genus has been previously reported to contain a variety of phenolic compounds [32]. In this study, we evaluated the TPC, TFC, and TCT contents of *A. armata* and *A. taxiformis* across their life stages through different extraction methods. The results are depicted in Table 1. TPC values recorded a similar trend in both UAE and CSE. The highest recorded TPC value was recorded in *A. armata* tetrasporophyte UAE (2.27 ± 0.13 mg GAE/g dw), closely followed by CSE (2.24 ± 0.05 mg GAE/g dw) for the same sample. The gametophyte stage of the *A. armata* displayed significantly lower TPC content compared to the diploid stage according to both extraction methods. While no similar comparative studies on the phenolic content of this species were identified, several studies have noted the biochemical differences in the stages of this alga’s life. Paul, de Nys and Steinberg [21] have recorded the higher presence of dibromoacetic acid and bromochloroacetic acid in the tetrasporophyte stage, but higher levels of bromoform and dibromochloromethane in the gametophyte, indicating complex biochemical alterations during the cyclic life of this species. On the other hand, *A. taxiformis* displayed a consistently lower TPC compared to its sister species, with significantly higher results obtained in the gametophyte stage of UAE. Appiah’s study [33] was the only other study identified that observed the phenolic content in different life stages in *A. taxiformis*. The authors have noted the higher TPC values of the gametophyte stage (1.19 ± 0.03 mg GAE/g) compared to the sporophyte (0.61 ± 0.07 mg GAE/g) in an ethyl acetate extraction. The differences in reported findings may stem from variations in extraction methodologies as well as differences in sourcing and habitat.

Other findings by Gao, Wang, Guo, Liu, Wu and Xiao [32] have reported the TPC of *A. taxiformis* up to 56.27 mg GAE/100 g dw depending on the extraction solvent and parameters. Nunes et al. [34] have reported similar values in the methanolic extract of *A. taxiformis* as 57.63 ± 3.92 mg GAE/100 dw, with other reports ranging between zero and 1.71 ± 0.13 g GAE/100 dw across different solvents [35]. We have previously been unable to observe the phenolic content of *A. taxiformis* gametophyte TPC in a refined extraction and reported an 8.57 ± 0.10 μg GAE/mg value in *A. armata* tetrasporophyte [28]. The variances in values may primarily stem from variations in cultivation habitats (UV exposure, temperature, and salinity), life stages, or extraction methodologies. Overall, the lack of a unified extraction protocol hinders meaningful comparisons of phenolic yield data reported across *Asparagopsis* species, with significant dissimilarities reported in the literature.

Phenolic compounds in macroalgae include a wide variety of secondary metabolites, with flavonoids as one of the major classes [36]. This study found clear differences in the TFC values across both life cycle stages and extraction methodologies. In *A. armata*, the gametophyte stage exhibited the highest flavonoid content in both CSE (1.00 ± 0.02 mg QE/g) and UAE (0.99 ± 0.05 mg QE/g) methodologies. A statistically significant decrease in the flavonoid content was recorded in the tetrasporophyte stage, reaching the lowest value of 0.23 ± 0.01 mg QE/g in CSE, potentially indicating a more pronounced presence of flavonoids within the haploid life stage. Analogous observations were recorded in *A. taxiformis*, with gametophyte reaching up to 0.74 ± 0.01 mg QE/g in CSE. As flavonoid constructure secondary metabolites, a significant increase in their production may indicate a higher stress response to the environmental challenges, metabolic needs, and other biotic or abiotic stressors. A comprehensive metabolic profiling of the changes during the life cycles is necessary to fully elucidate the biochemical pathways within the species [37].

To the best of our knowledge, no comparative reports of *Asparagopsis* flavonoid content between their life stages have been completed. Although Nunes, Valente, Ferraz, Barreto and De Carvalho [35] reported a range of 0.04 ± 0 g QE/100 g dw in water extraction to 24.25 ± 0.11 g QE/100 g dw in ethyl acetate extraction, showcasing the importance of the extraction solvent in flavonoid recovery in *A. taxiformis*. Reports of 73.44  ±  8.6 mg catechin E/g dw were recorded in a methanol–water extraction in Tunisia [38], and 0.006 mg catechin E/mL extract in a 50% ethanol extraction in Bangladesh [39]. The variation in the reports may reflect the effects of geographical variation in phytochemical composition, analytical procedures and units, and standard variations. Moreover, differences in the proteomic profile of *A. taxiformis* across different life stages have been observed, suggesting a diverse bioactive profile in various stages [40]. Acetone extract of *A. armata* has been reported to have 0.113 mg/g based on a quercetin calibration curve [41]. This study presents, to the best of our knowledge, the first comparative TFC analysis across life stages in *Asparagopsis* species.

In this project, the condensed tannin content of *Asparagopsis* species was evaluated using the vanillin–sulfuric acid assay, analyzing the rate of reaction between vanillin and any condensed tannins in the samples under acidic conditions to produce a red chromophore. However, no condensed tannins were observed in any of the samples under any of the extraction methods, in contrast to the observations in *A. taxiformis* collected from the Algerian West Coast [42] and Tunisia [38]. As secondary metabolites, tannin production and concentration may vary across habitats, cultivation methods, sunlight exposure, and other biotic and abiotic stressors [43,44]. Although, to the best of our knowledge, no reports of condensed tannins have been made in *A. armata*, and targeted analysis is needed to confirm their presence and concentration in this alga.

In principle, UAE is based on acoustic cavitation, leading to the formation and eventual collapse of a cavitation bubble in the extraction solvent due to the rapid changes in temperature and pressure. This may facilitate extraction through the instability of the plant cell wall matrix and increase in permeability, erosion, and the release of compounds into the solvent, particle fragmentation and the generation of smaller particle size, and facilitated solvent accessibility [45]. In this study, no universal advantages were observed in UAE compared to CSE in TPC and TFC, with an almost consistently higher values obtained in CSE, except in the tetrasporophyte stage of *A. armata* and the gametophyte stage of *A. taxiformis*. The lower observation in UAE may be attributed to the lower interaction period between the seaweed and solvent compared with CSE. Similar findings have been reported in *Ecklonia cava*, with higher total phenolic content observed in water and CSE extractions through 24 h of incubation compared to UAE for 6 and 12 h [46]. Moreover, Guandalini et al. [47] have also observed that UAE failed to significantly enhance phenolic compound recovery in mango peel compared to conventional approaches. Similar results were observed in mulberry pulp, where the UAE amplitude did not significantly affect the total phenolic compound recovery in extraction optimization [48].

Nevertheless, UAE can be considered a “green chemistry” extraction method that may decrease the extraction time with increased kinetics. However, cost–benefit analysis is necessary to determine whether UAE offers long-term advantages over conventional methods, particularly when considering equipment costs versus operator time [49]. Moreover, pilot studies will be essential to optimize extraction parameters such as time, power, and temperature [49,50], which may substantially improve extraction efficiency in a species-specific context, as observed by Gao, Wang, Guo, Liu, Wu and Xiao [32] in *A. taxiformis* extraction.

### 3.2. Estimation of Asparagopsis Antioxidant Potential

The *Asparagopsis* species possesses a complex matrix of bioactive phytochemicals, with different compounds capable of influencing antioxidative activity through various modes of action. Antioxidant assays evaluate the capacity of extracts to inhibit oxidative processes, with each method operating on distinct mechanisms of action and exhibiting differential chemical affinity toward specific constituents within complex mixtures. Therefore, the utilization of diverse assays to capture a more comprehensive profile of their antioxidant capacity is necessary [51]. Herein, seven in vitro antioxidant assays were employed to predict the radical scavenging ability, reducing power, and metal-chelating ability of the algal samples. The results are depicted in Table 2.

DPPH radical scavenging assay is a widespread, economic, and timely spectrophotometric method applied to measure antioxidant capacity in various foods, beverages, pure compounds, and plant extracts, including algae [52]. In this work, the effectiveness of the seaweed extract in neutralizing DPPH radical was evaluated. In *A. armata*, the gametophyte life stage displayed superior scavenging activity in both extraction methods, with 11.33 ± 0.36 and 10.85 ± 0.50 mg TE/g in CSE and UAE, respectively. The maturation-dependent scavenging activity may indicate a deeper physiological response to various internal or external stressors; however, a more detailed analysis of their metabolic profile is needed to identify potential indicators of antioxidative potential. *A. taxiformis* displayed a significantly lower scavenging potential compared to its sister species but presented no notable differences between their life stages in either extraction method. Although a significant increase in UAE was recorded in *A. taxiformis* gametophyte (from 7.35 to 9.14 mg TE/g) and *A. taxiformis* tetrasporophyte (from 6.45 to 10.04 mg TE/g), representing 24% and 56% improvements, respectively, these results may suggest ultrasound-enhanced extraction efficiency for *A. taxiformis*, with no improvements noted in *A. armata*, indicating a species-specific effect in extraction. Similar to our observations, *A. armata* displayed a higher DPPH potential in an ethanolic extraction than *A. taxiformis*; however, variations were observed in the aqueous extract depending on drying methodology [53].

Few studies have directly compared *Asparagopsis* species across life stages, but several have reported their antioxidant activities under different experimental conditions. The DPPH radical scavenging capacity of *A. taxiformis* has been reported to range from 24 to 52 mg AA/100 g in ethanolic extracts and 10 to 46 mg AA/100 g in aqueous extracts, depending on drying, storage conditions, and time [54]. The DPPH inhibitory concentration 50% (IC_50_) of this species was recorded at 0.40 (mg/mL) compared to 0.071 (mg/mL) in ascorbic acid [42]. Ktari, Ismail, Selmi, Hmani and Bour [38] also noted a range of 31.71 mg/mL to >100 EC50, depending on the extraction solvent. The differences in these observations likely arise from variation evaluations, extraction parameters, differences in geography, and results reporting formats [55]. While no comparative studies between life stages were identified in *A. armata*, previous reports have recorded a 23.6 ± 2.6% inhibition compared to Trolox-inhibition of DPPH at 89.7 ± 0.5% [11], with a EC_50_ of 862.45 ± 0.11 μg/mL for methanolic extract [56]. These results highlight the wide variability in DPPH assay outcomes for *Asparagopsis* species. It therefore suggests the need for a more standardized method of reporting data to enable more reliable comparisons between studies.

The ABTS radical scavenging assay is a low-cost method used to measure the total antioxidant capacity of plant extracts by measuring their ability to donate electron or hydrogen atoms to neutralize the stable ABTS radical cation [57], and can be used in completion to DPPH. *A. armata* gametophyte showed the highest ABTS scavenging activity across both extraction methodologies, yielding 13.85 ± 0.76 AAE/g in CSE and 15.62 ± 0.06 mg AAE/g in UAE, with reductions in the tetrasporophyte stage. *A. taxiformis* gametophyte had moderate and consistent values of ~11.6 mg AAE/g across both extraction methods, while *A. taxiformis* tetrasporophyte displayed a non-significant increase in activity under UAE extraction (12.20 ± 1.12 mg AAE/g). In a survey of the effects of drying techniques in Portugal, the ABTS potential of ethanolic extract of *A. taxiformis* displayed a non-significant edge over their sister species, with varying observations in the aqueous extract [53]. The ethanolic extract of *A. armata* gametophyte has been recorded at 0.24 mg/mL IC_50_ [33], while *A. taxiformis* was noted between 0.84 and 4.40 in aqueous extract and 0.26 and 2.46 (mmol TE/100 g dw) in ethanolic extract, depending on storage time and conditions [54]. The same species was recorded between 1.67 and4.78 mM TE/g dw in different extraction parameters [32]. Differences in extraction procedures, seasonal timing, habitat, and environmental conditions may account for these variations.

The hydroxyl radical (OH) is one of the most reactive and damaging reactive oxygen species in biological systems, exhibiting non-selective reactivity toward critical biomacromolecules, including DNA, proteins, and lipids [58]. In this study, the seaweeds’ ability to neutralize OH radicals generated through the Fenton reaction was observed. A narrow range of activity was recorded for all samples, with no statistically significant differences in activity between gametophyte and tetrasporophyte forms of either species under CSE or UAE. These findings may indicate that the compounds responsible for hydroxyl radical scavenging in both *Asparagopsis* species are effectively extracted by both methods. Under UAE conditions, a significant difference was observed between *A. armata* tetrasporophyte (44.52 ± 0.16 mg AAE/g) and its gametophyte (41.90 ± 0.5 mg AAE/g), whereas no significant differences were detected between life cycle stages of *A. taxiformis*. A higher rate of activity in *A. armata* was observed in our earlier study, with no recordable potential noted in *A. taxiformis* [28], most likely stemming from the variation in extraction methodologies. Overall, across the different assays, *A. armata* demonstrated superior scavenging potential compared to its sister species, indicating a higher antioxidative capacity in these tests. However, to make a comprehensive assessment of their overall potential, it is important to consider additional assay methods conducted, as different modes of action may infer various findings.

In addition to their scavenging capabilities, antioxidants can also reduce the valent elements in their environment, leading to lowered redox potential. Therefore, it is necessary to gauge the seaweeds’ reducing capacity to better comprehend their overall potential. To this end, three reducing assays were carried out: FRAP, which is based on the extracts’ ability to reduce ferric ions (Fe^3+^) to ferrous ions (Fe^2+^) [59]; RPA, which measures the reducing potential of potassium ferricyanide (K_3_[Fe(CN)_6_]) to potassium ferrocyanide (K_4_[Fe(CN)_6_]) [60]; and TAC, which assesses the reduction of the phosphomolybdate Mo(VI) complex to the reduced molybdenum Mo(V) complex [61].

In this study, *A. armata* gametophyte exhibited significantly higher ferric reducing power compared to all other samples, with a non-significantly higher potential obtained through UAE in both life stages. Similar recordings were obtained in RPA across life stages, with higher values obtained through UAE. Consistent recordings were noted in *A. taxiformis* across extraction methodologies and life stages in FRAP (0.89–0.94 TE mg/g), with a significantly higher potassium ferricyanide (0.07 ± 0.01 compared to 0.04 ± 0.01 TE mg/g) reducing potential in tetrasporophyte samples. Nunes, Ferraz, Valente, Barreto and Pinheiro de Carvalho [34] noted the FRAP content of the Portuguese *A. taxiformis* as 71.69 ± 2.11 mg AAE/100 g dw, similar to our observations, although the variation in standard limits the direct comparability. Moreover, a 67% ferric reducing antioxidant activity in chloroform extract of the same species collected from South India was recorded at 500 µg/mL concentration, similar to the utilized ascorbic acid standard [13]. A range of 1.30–4.60 mM Fe(II)/g dw using FeSO_4_ as the standard has also been reported in different extraction parameters [32]. In *A. armata*, previous reports of 0.145 mg vitamin C equivalent/mL FRAP activity have been recorded in Algerian seaweed [41]. As no other life-stage studies could be identified and given the considerable variation in extraction methods and reporting approaches across the available literature, direct comparisons remain difficult. Nonetheless, the findings indicate that *Asparagopsis* exhibits measurable iron-reducing potential.

In contrast to the ferric base methods, *A. armata* tetrasporophyte displayed a significantly higher potential through TAC assay in both extraction methods, with CSE achieving significantly higher results (5.62 ± 0.35 in vs. 4.88 ± 0.18 TE mg/g). A similar pattern was also noted in *A. taxiformis* in the life stage (3.53 ± 0.24 compared to 2.55 ± 0.12 TE mg/g in CSE). *A. armata* displayed a relatively higher potential than *A. taxiformis*, indicating a possible stronger presence of compounds capable of reducing molybdenum. In general, UAE extraction tended to reduce TAC values compared to CSE, indicating a possible breakdown of reducing compounds during ultrasonication or a more comprehensive extraction through conventional methods. Overall, *A. taxiformis* revealed relatively lower reducing power potential than *A. armata*, displaying a similar pattern observed in the scavenging potential. The final chelating assay described below will allow for a more roundabout understanding between the species.

In the FICA assay, the seaweed extracts act as metal-chelating agents by binding ferrous ions (Fe^2+^) and limiting their activity in the reaction mixture, thereby inhibiting the formation of the ferrozine–Fe^2+^ complex, which can be measured colorimetrically [62]. Under CSE, *A. armata* tetrasporophyte showed the highest chelating activity (0.77 ± 0.02 mg EDTAE)/g), significantly higher than all other samples. A similar decrease in chelating activity was noted in the gametophyte stage in UAE. In contrast, *A*. *taxiformis* preserved a similar activity in both extractions, with the gametophyte stage having a significantly higher potential, in contrast to its sister species, indicating a complex species-specific metabolomic profile across the *Asparagopsis* life cycle. Overall, *A. armata* displayed an overall higher chelating activity, as observed in our earlier studies of their refined extracts [28].

### 3.3. Relative Antioxidant Capacity Index (RACI) of Asparagopsis

As previously noted, no single assay can comprehensively capture the overall antioxidant potential of a sample. Moreover, the results obtained from different assays are not always directly comparable, as they rely on different standards, detection methods, and chemical principles. To overcome these inconsistencies and generate a unified evaluation, a dimensionless, standardized scoring approach is required. Therefore, we employed the RACI method to integrate the assay outcomes and provide a consolidated measure of the samples’ antioxidant potential [27]. Among the samples, CSE-extracted *A. armata* reached the highest overall score in the tetrasporophyte (0.77), followed by the gametophyte stage (0.64), displaying the superior antioxidative potential in *A. armata* tetrasporophyte. The UAE of this species received the next highest ranking, while the CSE *A. taxiformis* sample showed the lowest overall potential. From these results, *A. armata* demonstrates a generally higher antioxidant capacity than its sister species; however, more targeted bioactivity assays are required to corroborate the biological relevance of these findings. Furthermore, detailed characterization of their phenolic profiles may help clarify and predict the broader bioactivities expected from these samples.

### 3.4. Correlation of Polyphenols and Antioxidant Activities

Pearson’s correlation was used to assess the strength of associations between antioxidant capacities determined by different methodologies, as well as their relationships with phenolic and flavonoid contents in *Asparagopsis* extracts, with the results depicted in Table 3. This study showed a strong positive correlation between TPC and TAC, with the correlation coefficient (*r*) recorded as 0.90, indicating the possible role of phenolic compounds in reducing phosphomolybdate complex. Moreover, the modest correlation between this assay and hydroxyl radical scavenging and metal-chelating activity may also point to the role of phenolic compounds as antioxidants in *Asparagopsis*. Similarly, the flavonoid portion recorded a robust correlation with ferric ion reducing activity, with a strong positive association with FRAP and RPA assays. As both assays quantify reducing capacity via electron-transfer mechanisms, this relationship may suggest that flavonoids in *Asparagopsis* contribute substantially to its electron-donating ability. On the other hand, the negative association in hydroxyl radical scavenging activities and metal-chelating assay recorded may indicate the role of other components in the presentation of overall *Asparagopsis* antioxidant activity. FRAP and RPA assays recorded the strongest positive correlation in this study (*r*= 93, *p* < 001), with similar findings reported in Jackfruit [63]. DPPH and ABTS radical scavenging measures were also moderately similar, as they both measure the scavenging potential in different mechanisms, as recorded in previous studies [26]. Overall, the interplay between the antioxidant mechanisms and the phenolic profile of *Asparagopsis* species reveals a complex pattern of associations that warrants further investigation.

### 3.5. Distribution of Phenolic Compounds Across Asparagopsis Species, Life Stages, and Extraction Methodology

**Figure 1:**
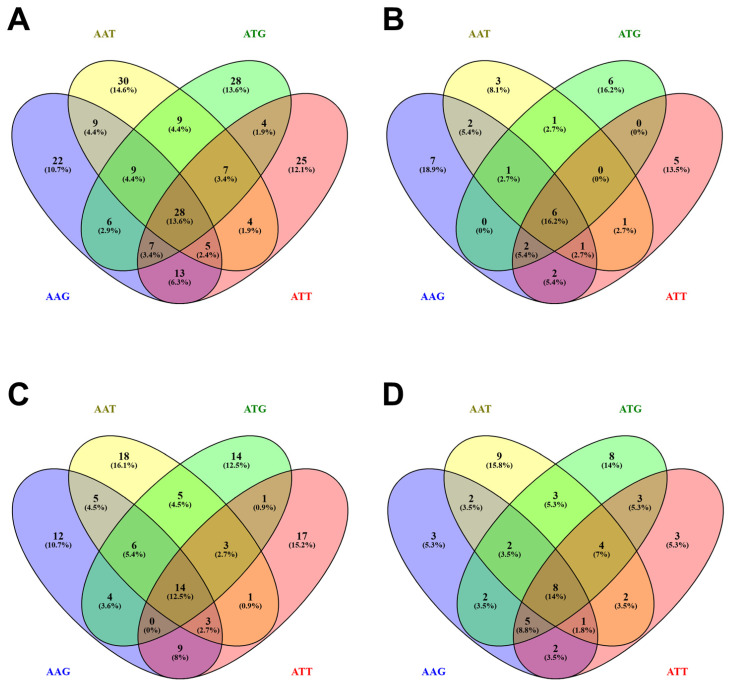
Venn diagram of the distribution of phenolic compounds present in *Asparagopsis* species. As labeled in each graph, distinct colors represent the seaweed species according to their life stages, with overlapping regions indicating shared compounds. AAT: *Asparagopsis armata* Tetrasporophyte; AAG: *Asparagopsis taxiformis* Gametophyte; ATG: *Asparagopsis taxiformis* Tetrasporophyte; ATT: *Asparagopsis armata* Tetrasporophyte. (**A**) shows the relation of total polyphenols in the *Asparagopsis* species; (**B**) shows the relation of phenolic acids in *Asparagopsis* species; (**C**) shows the relation of flavonoids in *Asparagopsis* species; and (**D**) shows the relation between the other polyphenols in *Asparagopsis* species.

**Figure 2:**
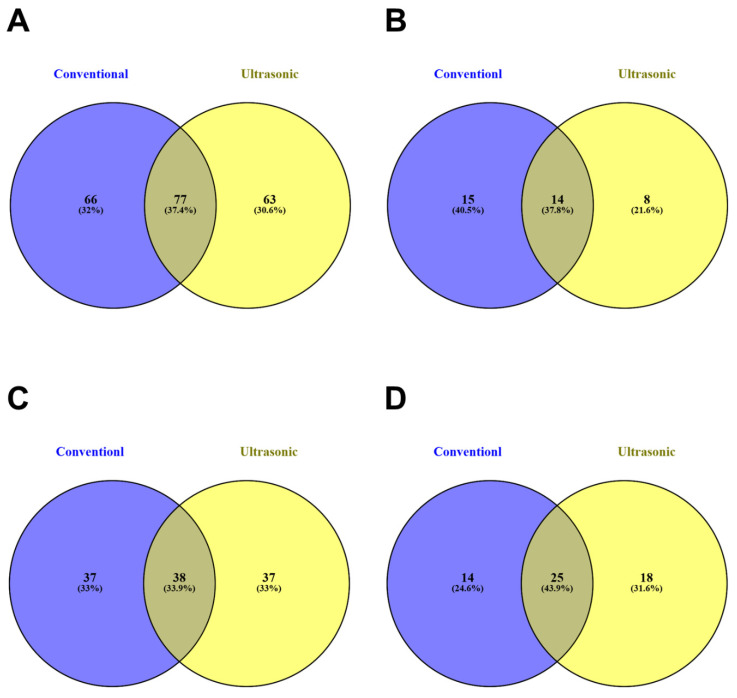
Venn diagram of the distribution of phenolic compounds obtained through conventional solvent extraction and ultrasonic assisted extraction. As labeled in each graph, distinct colors represent the extraction methodology, with overlapping regions indicating shared compounds. (**A**) shows the relation of total polyphenols in the *Asparagopsis* species; (**B**) shows the relation of phenolic acids in *Asparagopsis* species; (**C**) shows the relation of flavonoids in *Asparagopsis* species; and (**D**) shows the relation between the other polyphenols in *Asparagopsis* species.

To better comprehend the effects of species, life stage, and extraction method, Venn diagrams of the screened phenolic metabolites were drafted to visually observe the differences in the phenolic profile of the samples. Overall, 206 compounds were screened across all samples. As observed in Figure 1A, 88 compounds were shared in both species, with 61 unique compounds observed in *A. armata* and 57 in *A. taxiformis*, suggesting the presence of core phenolic metabolites at the genus level. Although more than 57% of the screened compounds were uniquely identified in the species, pointing to the species-specific dynamics and metabolism that occurs in the macroalgal life in response to biotic and abiotic stresses, cultivation conditions, and life stage, similar pattern was documented in the growth stages, with 91 shared compounds present across life stages, and more than 55 compounds only emerging in either stage. Similar metabolomic changes have also been noted previously [20]; however, a full metabolomic profile is essential to realize the complexities of changes across the systems. Figure 2A depicts the comparisons of all phenolic compounds between extraction methodologies, with less than 40% of the compounds obtained through both extraction processes, underlying the influence of extraction on biological function, as noted previously [64]. Moreover, in contrast to the other groupings, the unique phenolic acids obtained through CSE were almost twice as many as those obtained through the UAE, possibly indicating an influence on the observed variation in the phenolic content and antioxidant potentials. To the best of our knowledge, no studies to date have examined *Asparagopsis* using comparative analyses across extraction approaches or developmental stages.

### 3.6. LC-ESI-QTOF-MS/MS Characterization of Phenolic Compounds

LC–ESI–QTOF–MS/MS remains one of the most reliable and routinely applied approaches for screening and characterizing bioactive metabolites. In the present work, phenolic compounds from genus *Asparagopsis* were profiled in both positive and negative ionization modes using the LC–ESI–QTOF–MS/MS platform using Mass Hunter workstation software version (B.03.01) and the Personal Compound Database and Library (PCDL). To ensure high confidence in compound annotation, only records with a mass error of less than ±5 ppm and a PCDL matching score above 80 were retained for subsequent MS/MS-based confirmation and *m*/*z* characterization. Across the samples, 24 phenolic compounds were tentatively characterized, including (9) phenolic acids, made up of (2) hydroxybenzoic acids, (6) hydroxycinnamic acids, and (1) hydroxyphenylepropanoic acid. Additionally, (15) flavonoids were putatively characterized, including (5) flavonols, (4) flavanones, (4) flavones, and (2) flavanols. A summary of the data is provided in Table 4.

Compound **1** was tentatively identified as ellagic acid based on its [M-H]^−^ ion at *m*/*z* 300.9960 and MS/MS fragment ions at *m*/*z* 284, 229, and 201 product ions. Ellagic acid was detected in ATT extracted using UAE. Compound **2**, as an ellagic acid derivative, was also putatively recognized in ATG extracted using CSE. Ellagic acid has displayed promising potential in the management of chronic diseases, including hepatoprotective, anti-carcinogenic, and anti-diabetic properties [65], and has been previously observed in red seaweeds [66]. Compound **5** was tentatively identified based on the precursor ion at *m*/*z* 397.1149 and product ions at *m*/*z* 233 and 179 as 3-Sinapoylquinic acid, in both development stages of *A. armata*. 3-Sinapoylquinic acid is the result of the reversible esterification of quinic acid with sinapic acid [67], with a widespread potential as a health agent [68]. This compound has also been recorded in *Gracilaria edulis* collected from the Red Sea in Egypt [66].

Furthermore, compound **8**, 4,5-Dicaffeoylquinic acid, was identified at [M-H]^−^ at *m*/*z* 515.1159, with product ions at 353, due to the possible loss of caffeoyl group (162 Da), and 335, a water molecule (18 Da) [69]. The *m*/*z* 191 and 179 may also correspond with the fragmentation of quinic and caffeic acid moieties [70]. This compound was detected in both stages of *A. armata* and the tetrasporophyte stage of *A. taxiformis*. A study by Jang et al. [71] showed that 4,5-Dicaffeoylquinic acid exerts anti-inflammatory effects by suppressing carrageenan-induced edema in in vivo trials and could be a potential natural alternative to non-steroidal anti-inflammatory drugs.

In this study, several flavonoids were also provisionally identified. Compound **10** displayed an observed *m*/*z* at 289.0714 in negative ionization mode, preliminary recorded as (−)-epicatechin, and further confirmed by the MS/MS experiment, which displayed a loss of product ion at *m*/*z* 245, 205, and 179. This compound has been previously recorded in *A. taxiformis* in China, determined by Ultra-High-Performance Liquid Chromatography coupled with Triple Quadrupole Mass Spectrometry (UHPLC-QQQ-MS) [72]; however, it was only observed in *A. armata* tetrasporophyte UAE in this study. This compound has been the focus of many studies, with proposed anti-inflammatory, anti-diabetic, and neuroprotective properties, with promising observations in skeletal muscle enhancement and cerebrovascular health [73,74]. Another key flavonol is quercetin, with extensive reported bioactivities [75]. Quercetin derivatives were detected across all samples, consistent with earlier reports documenting this compound and its derivatives in diverse seaweed taxa [76,77]. Overall, this study tentatively identified 24 phenolic constituents in the genus *Asparagopsis*, with 17 compounds in *A. armata* and 14 in *A. taxiformis*. Although these observations remain putative pending targeted confirmation, many of these compounds have been associated with diverse biological activities, underscoring the need for further verification and functional assessment.

## 4. Conclusions

In this work, the phenolic profile and antioxidant potential of *Asparagopsis* were evaluated in *A. armata* and *A. taxiformis* in both life stages. *A. armata* recorded a more pronounced potential compared to its sister species. Additionally, ultrasound-assisted extraction did not record a universal advantage but displayed species- and life stage-specific potentials in certain assays. Correlation analysis provided insights into the relationships between total phenolic content, flavonoid content, and various antioxidant assays. Moreover, 24 compounds were tentatively recorded across the samples, with different life stages possessing specific phenolic profiles. However, this study is limited to the analysis of two developmental stages of seaweed using 80% ethanol as the extraction solvent. A more comprehensive analysis of all three life phases with different solvents will enhance the overall knowledge of seaweed metabolites, paving the way for possible targeted analysis. Overall, *Asparagopsis* samples contain a multitude of bioactive compounds, with some specific to a species/life stages, and more targeted analysis is required to fully capture the full potential of this marine resource.

## Figures and Tables

**Table 1:** Total phenolic content and total flavonoids of *Asparagopsis* species at different lifecycle stages using conventional and ultrasound-assisted extraction methods.

Sample	Life Stage	TPC mg GAE/g	TFC mg QE/g
Conventional solvent extraction			
*A. armata*	tetrasporophyte	2.24 ± 0.05 ^a^	0.23 ± 0.01 ^e^
	gametophyte	1.28 ± 0.03 ^b^	1.00 ± 0.02 ^a^
*A. taxiformis*	tetrasporophyte	0.48 ± 0.02 ^c,d^	0.54 ± 0.02 ^c^
	gametophyte	0.46 ± 0.01 ^d^	0.74 ± 0.01 ^b^
Ultrasonic-assisted extraction			
*A. armata*	tetrasporophyte	2.27 ± 0.13 ^a^	0.51 ± 0.01 ^c^
	gametophyte	1.24 ± 0.01 ^b^	0.99 ± 0.05 ^a^
*A. taxiformis*	tetrasporophyte	0.46 ± 0.03 ^d^	0.39 ± 0.01 ^d^
	gametophyte	0.58 ± 0.05 ^c^	0.53 ± 0.01 ^c^

Results are expressed in mean ± standard deviation (SD) in triplicate. Means that do not share a letter (^a,b,c,d,e^) within the same assay are significantly different (*p* < 0.05). GAE: Gallic acid equivalent; QE: Quercetin equivalent.

**Table 2:** Antioxidant potential of four red seaweed varieties extracted using conventional and ultrasonic-assisted methods.

Sample	Life Stage	DPPH TE mg/g	ABTS AAE mg/g	OH^−^RSA AAE mg/g	FRAP TE mg/g	RPA TE mg/g	TAC TE mg/g	FICA EDTAE mg/g	RACI
Conventional solvent extraction									
*A. armata*	tetrasporophyte	8.93 ± 0.68 ^c,d^	12.79 ± 0.64 ^b,c^	44.69 ± 0.51 ^a^	1.71 ± 0.15 ^b^	0.05 ± 0.01 ^e^	5.62 ± 0.35 ^a^	0.77 ± 0.02 ^a^	0.77
	gametophyte	11.33 ± 0.36 ^a^	13.85 ± 0.76 ^a,b^	42.88 ± 0.66 ^a,b^	3.32 ± 0.23 ^a^	0.19 ± 0.01 ^b^	3.59 ± 0.15 ^c^	0.49 ± 0.03 ^b^	0.64
*A. taxiformis*	tetrasporophyte	6.45 ± 0.29 ^f^	10.20 ± 0.55 ^d^	42.89 ± 1.24 ^a,b^	0.94 ± 0.07 ^c^	0.07 ± 0.01 ^d^	3.53 ± 0.24 ^c^	0.40 ± 0.01 ^c^	−0.72
	gametophyte	7.35 ± 0.26 ^e,f^	11.65 ± 1.02 ^c,d^	43.09 ± 0.72 ^a,b^	0.92 ± 0.09 ^c^	0.04 ± 0.01 ^e^	2.55 ± 0.12 ^d^	0.50 ± 0.05 ^b^	−0.59
Ultrasonic-assisted extraction									
*A. armata*	tetrasporophyte	7.99 ± 0.16 ^d,e^	10.73 ± 0.30 ^c,d^	44.52 ± 0.16 ^a^	1.90 ± 0.11 ^b^	0.08 ± 0.01 ^c^	4.88 ± 0.18 ^b^	0.54 ± 0.04 ^b^	0.33
	gametophyte	10.85 ± 0.50 ^a,b^	15.62 ± 0.06 ^a^	41.90 ± 0.51 ^b^	3.62 ± 0.37 ^a^	0.21 ± 0.01 ^a^	3.46 ± 0.26 ^c^	0.36 ± 0.01 ^c^	0.54
*A. taxiformis*	tetrasporophyte	10.04 ± 0.48 ^b,c^	12.20 ± 1.12 ^b,c,d^	43.07 ± 0.40 ^a,b^	0.89 ± 0.16 ^c^	0.07 ± 0.01 ^d^	2.07 ± 0.15 ^e^	0.39 ± 0.02 ^c^	−0.45
	gametophyte	9.14 ± 0.29 ^c^	11.57 ± 0.78 ^c,d^	42.44 ± 0.80 ^b^	0.89 ± 0.06 ^c^	0.04 ± 0.01 ^e^	1.94 ± 0.11 ^e^	0.55 ± 0.04 ^b^	−0.52

Results are expressed in mean ± standard deviation (SD) in triplicate. Values that do not share a letter (^a,b,c,d,e,f^) within the same assay are significantly different (*p* < 0.05). TE: Trolox equivalent; EDTAE: Ethylenediaminetetraacetic acid equivalent; AAE: Ascorbic acid equivalent; DPPH, 2,2-diphenyl-1-picrylhydrazyl radical scavenging activity; FRAP, ferric reducing antioxidant power; ·OH, hydroxyl radical scavenging activity; ABTS, 2,2′-Azino-bis(3-ethylbenzothiazoline-6-sulfonic acid) radical scavenging activity; FICA, ferric ions chelating activity; TAC, total antioxidant capacity; RPA, reducing power activity.

**Table 3:** Pearson’s correlation coefficients between phenolic content (TPC and TFC) and antioxidant activities (DPPH, FRAP, ABTS, FICA, TAC, OH, and RPA).

	TPC	TFC	DPPH	FRAP	OH	ABTS	FICA	TAC
TFC	−0.20							
DPPH	0.14	0.44 *						
FRAP	0.44 *	0.71 **	0.68 **					
OH	0.61 **	−0.58 **	−0.24	−0.23				
ABTS	0.16	0.55 **	0.76 **	0.78 **	−0.34			
FICA	0.60 **	−0.54 **	−0.10	−0.16	0.64 **	−0.16		
TAC	0.90 **	−0.24	−0.12	0.34	0.59 **	0.04	0.57 **	
RPA	0.16	0.81 **	0.69 **	0.93 **	−0.41 *	0.75 **	−0.45 *	0.09

Asterisks indicate statistical significance (* *p* < 0.05; ** *p* < 0.01).

**Table 4:** Characterization of phenolic compounds from eight *Asparagopsis* samples through LC-ESI-QTOF-MS^2^.

No.	Proposed Compounds	Molecular Formula	RT (min)	Mode of Ionization	Molecular Weight	Theoretical (*m*/*z*)	Observed (*m*/*z*)	Mass Error (ppm)	MS/ MS	Samples
	**Phenolic acids**									
	Hydroxybenzoic acid									
1	Ellagic acid	C_14_H_6_O_8_	54.797	[M-H]^−^	302.0039	300.9966	300.9960	−2.0	284, 229, 201	ATT-U
2	Ellagic acid acetyl-arabinoside	C_21_H_16_O_13_	55.990	[M-H]^−^	476.0572	475.0499	475.0480	−4.0	301	ATG-C
Hydroxycinnamic acids										
3	*p*-Coumaric acid 4-*O*-glucoside	C_15_H_18_O_8_	53.675	[M-H]^−^	326.1012	325.0939	325.0932	−2.2	163	ATG-C
4	Ferulic acid 4-*O*-glucuronide	C_16_H_18_O_10_	54.120	[M-H]^−^	370.0901	369.0828	369.0815	−3.5	178, 193	AAG-C
5	3-Sinapoylquinic acid	C_18_H_22_O_10_	54.288	[M-H]^−^	398.1222	397.1149	397.1134	−3.8	233, 179	* AAT-C, AAG-C
6	5-Feruloylquinic acid	C_17_H_20_O_9_	54.609	[M-H]^−^	368.1086	367.1013	367.1020	1.9	298, 288, 192, 191	AAT-U
7	Rosmarinic acid	C_18_H_16_O_8_	54.714	[M-H]^−^	360.0862	359.0789	359.0796	1.9	179	AAT-C
8	4,5-Dicaffeoylquinic acid	C_25_H_24_O_12_	54.844	** [M-H]^−^	516.1244	515.1171	515.1159	−2.3	353, 335, 191, 179	* AAT-U, AAG-C, ATT-C, AAG-U
Hydroxyphenylpropanoic acids										
9	Dihydroferulic acid 4-sulfate	C_10_H_12_O_7_S	4.045	[M-H]^−^	276.0309	275.0236	275.0225	−4.0	195, 151, 177	AAT-U
**Flavonoids**										
Flavonols										
10	(−)-Epicatechin	C_15_H_14_O_6_	4.045	[M-H]^−^	290.0785	289.0712	289.0714	0.7	245, 205, 179	AAT-U
11	Quercetin 3-*O*-(6″-malonyl-glucoside)	C_24_H_22_O_15_	45.093	[M+H]^+^	550.0943	551.1016	551.1019	0.5	303	* ATG-C, AAG-C, AAT-C, ATT-C, AAT-U, AAG-U, ATG-U, ATT-U
12	Quercetin 3-*O*-xyloside	C_20_H_18_O_11_	45.506	[M-H]^−^	434.0840	433.0767	433.0788	4.8	301	ATT-U
13	Quercetin 3-*O*-glucosyl-xyloside	C_26_H_28_O_16_	53.944	[M-H]^−^	596.1388	595.1315	595.1312	−0.5	265, 138, 116	AAG-C
14	Quercetin 3′-sulfate	C_15_H_10_O_10_S	54.089	[M-H]^−^	381.9975	380.9902	380.9892	−2.6	301	* ATT-C, AAT-C, AAG-U, ATG-U, ATT-U
Flavanones										
15	Hesperetin 3′-sulfate	C_16_H_14_O_9_S	54.717	[M-H]^−^	382.0375	381.0302	381.0303	0.3	301, 286, 257, 242	ATT-U
16	Hesperetin 5,7-*O*-diglucuronide	C_28_H_30_O_18_	4.045	[M-H]^−^	654.1418	653.1345	653.1321	−3.7	477, 301, 286, 242	AAT-U
17	Hesperetin 3′-*O*-glucuronide	C_22_H_22_O_12_	48.755	[M-H]^−^	478.1128	477.1055	477.1033	−4.6	301, 175, 113, 85	ATG-U
18	Naringin 4′-*O*-glucoside	C_33_H_42_O_19_	54.393	[M-H]^−^	742.2304	741.2231	741.2229	−0.3	433, 271	AAG-C
Flavones										
19	Nobiletin	C_21_H_22_O_8_	53.703	** [M+H]^+^	402.1352	403.1425	403.1428	0.7	388, 373, 355, 327	* ATG-U, ATT-U, AAG-U, AAT-U, ATG-C, AAG-C
20	Luteolin 7-*O*-(2-apiosyl-glucoside)	C_26_H_28_O_15_	55.528	[M+H]^+^	580.1416	581.1489	581.1479	−1.7	419, 401, 383	ATG-C
21	Apigenin 7-*O*-(6′’-malonyl-apiosyl-glucoside)	C_29_H_30_O_17_	57.684	[M-H]^−^	650.1488	649.1415	649.1413	−0.3	605	* AAT-U, ATG-U
22	Apigenin 7-*O*-diglucuronide	C_27_H_26_O_17_	57.701	[M-H]^−^	622.1184	621.1111	621.1111	0.0	269	* ATT-U, AAG-U
Flavonols										
23	(+)-Catechin 3-*O*-gallate	C_22_H_18_O_10_	54.306	** [M-H]^−^	442.0879	441.0806	441.0812	1.4	289, 169, 125	* AAG-C, AAT-C, ATT-C, ATG-U, ATT-U
24	Isorhamnetin 4′-*O*-glucuronide	C_22_H_20_O_13_	4.045	** [M-H]^−^	492.0892	491.0819	491.0813	−1.2	315, 300, 272, 255	* AAT-U, AAG-U

* Indicates that compound was detected in more than one sample, with data presented for the assigned sample. ** Indicates that compound was detected in both positive [M+H]^+^ and negative modes [M-H]^−^. *Asparagopsis* samples were represented by abbreviations: *Asparagopsis armata* tetrasporophyte-Conventional (AAT-C), *Asparagopsis armata* Gametophyte-Conventional (AAG-C), *Asparagopsis taxiformis* Gametophyte-Conventional (ATG-C), *Asparagopsis taxiformis* Tetrasporophyte (ATT-C), *Asparagopsis armata* Tetrasporophyte-Ultrasonic (AAT-U), *Asparagopsis armata* Gametophyte-Ultrasonic (AAG-U), *Asparagopsis taxiformis* Gametophyte-Ultrasonic (ATG-U), and *Asparagopsis taxiformis* Tetrasporophyte-Ultrasonic (ATT-U).

## Data Availability

The original contributions presented in this study are included in the article/Supplementary Material. Further inquiries can be directed to the corresponding author.

## Supplementary Materials

The following supporting information can be downloaded at: https://www.mdpi.com/article/10.3390/antiox15020273/s1, Figure S1: Normal Q–Q plots of model residuals used to evaluate the normality assumption for each phenolic and antioxidant assay.
