# Supplementary material for: Phenolic Characterization and Comparative Antioxidant Profiling of Australian Asparagopsis armata and A. taxiformis Across Their Developmental Stages
Source: Antioxidants (Basel). 2026 Feb 23;15(2):273. doi: 10.3390/antiox15020273 (PMC12937925; doi:10.3390/antiox15020273)
Supplement: Supplementary file 1 [file antioxidants-15-00273-s001.zip › antioxidants-4091011-supplementary.pdf]

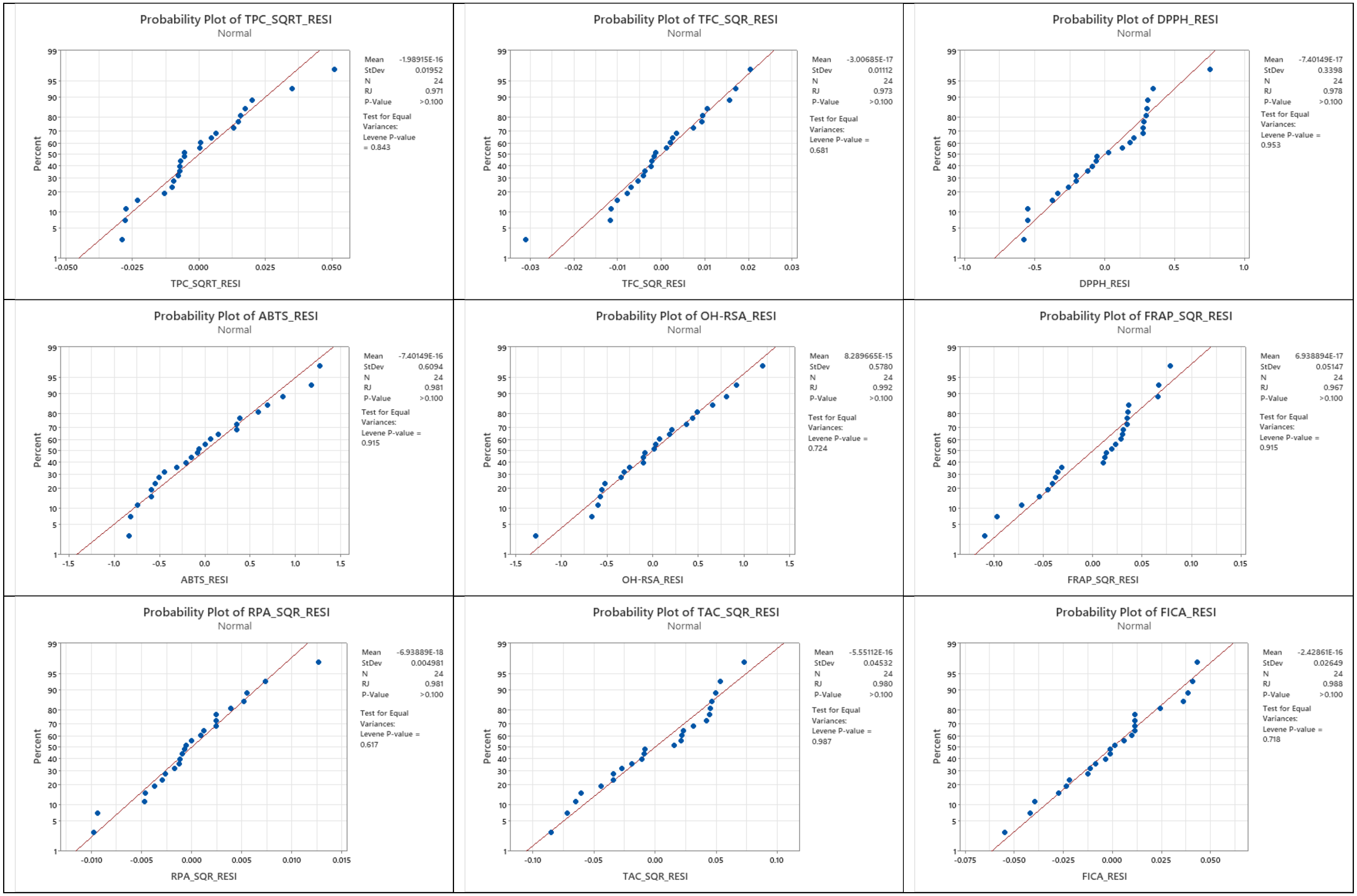

Supplementary Figure S1. Normal Q–Q plots of model residuals used to evaluate the normality assumption for each phenolic and antioxidant assay. Residual normality was assessed using the Ryan–Joiner test ($p < 0.05$). If residuals deviated from normality, a square-root (SQRT) transformation was applied and the

assumptions were re-evaluated. The red diagonal line represents the theoretical reference line for perfect normality. Homogeneity of variances was assessed using Levene's test on the residuals for each assay, with the corresponding p value displayed on graph.
